# Supplementary figures and images for: Geographies of an Online Social Network
Source: PLoS One. 2015 Sep 11;10(9):e0137248. doi: 10.1371/journal.pone.0137248 (PMC4567269; doi:10.1371/journal.pone.0137248)

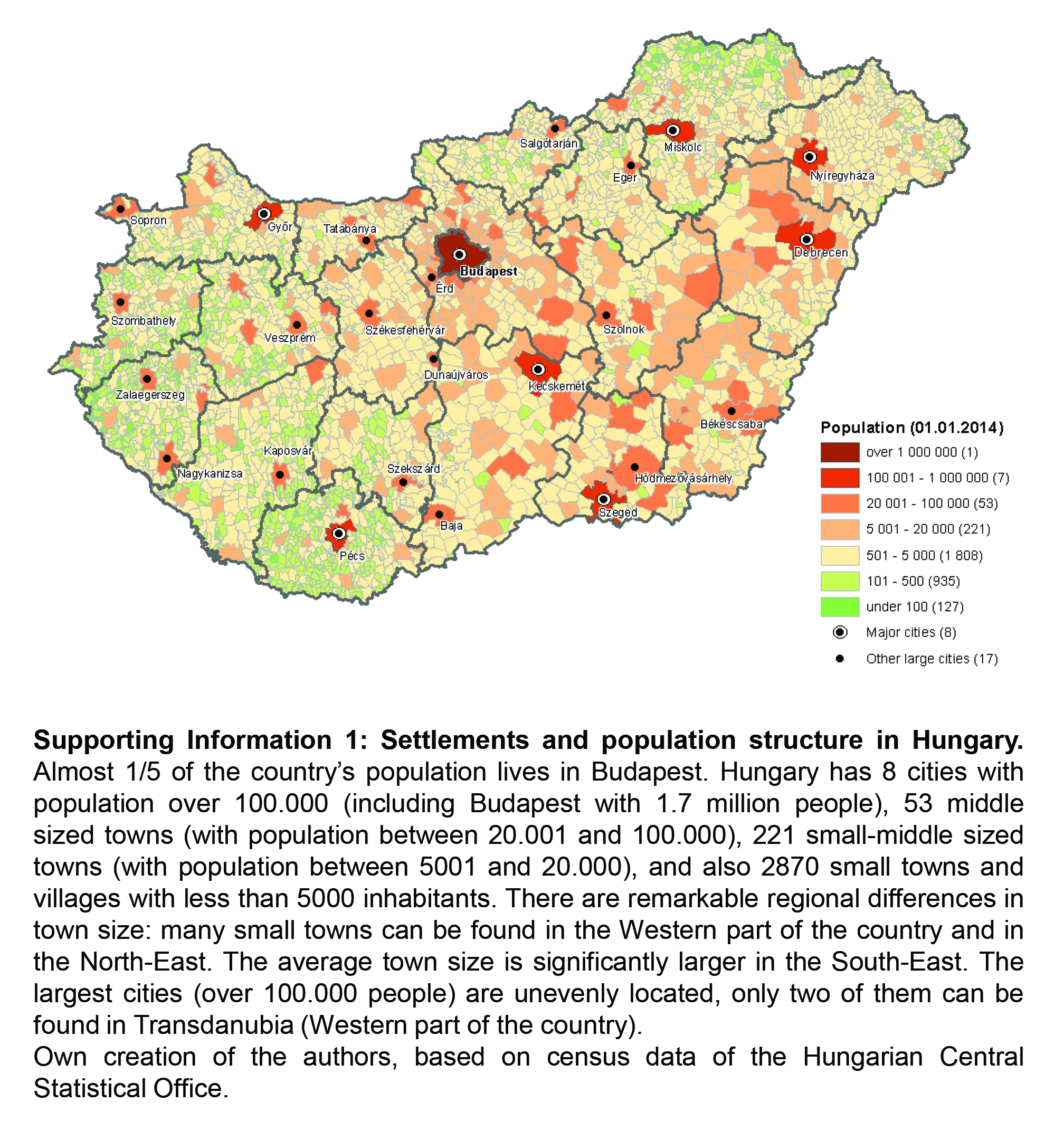

Supplement: S1 Fig — (TIF) [file pone.0137248.s001.tif]

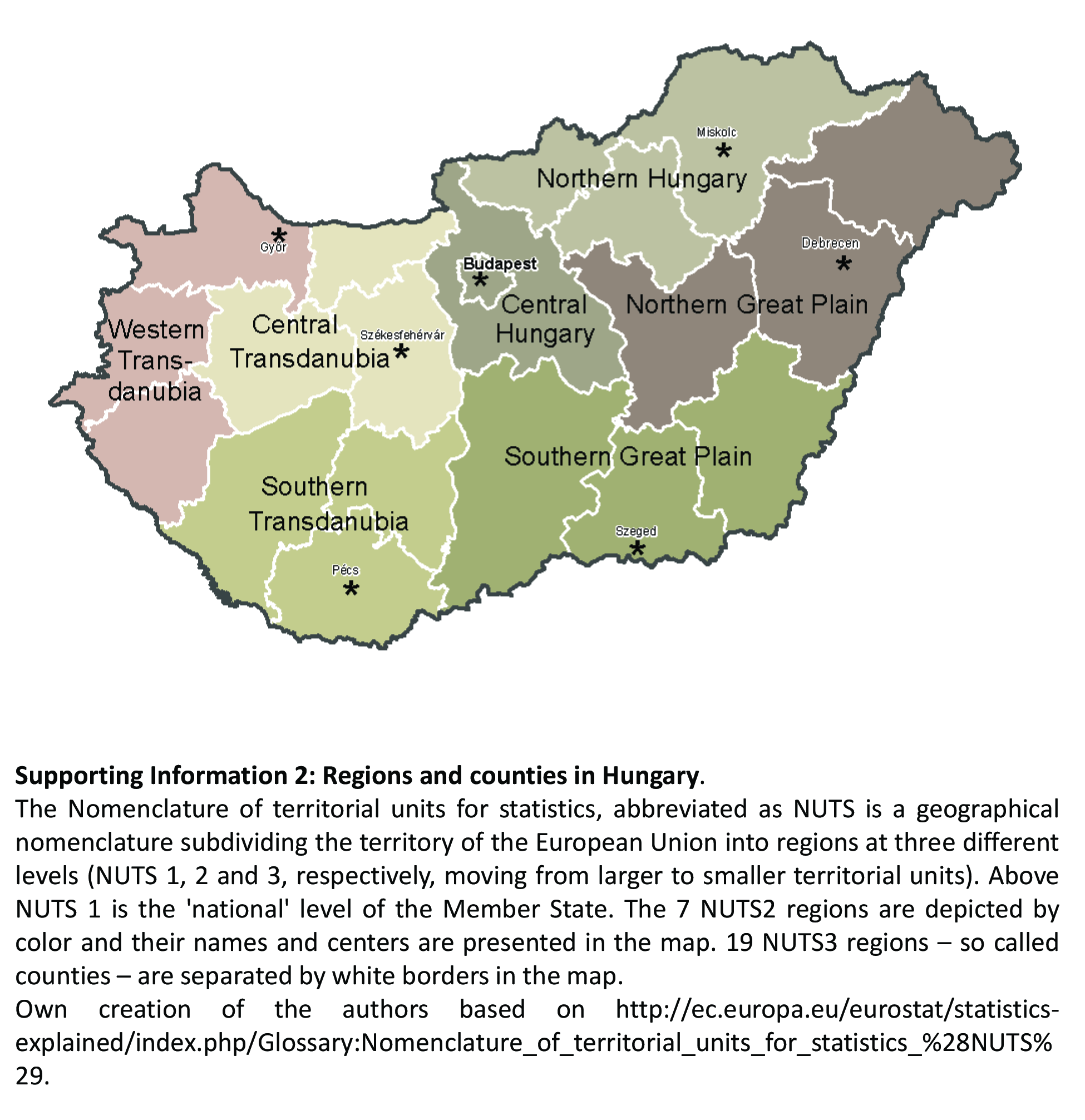

Supplement: S2 Fig — (TIF) [file pone.0137248.s002.tif]

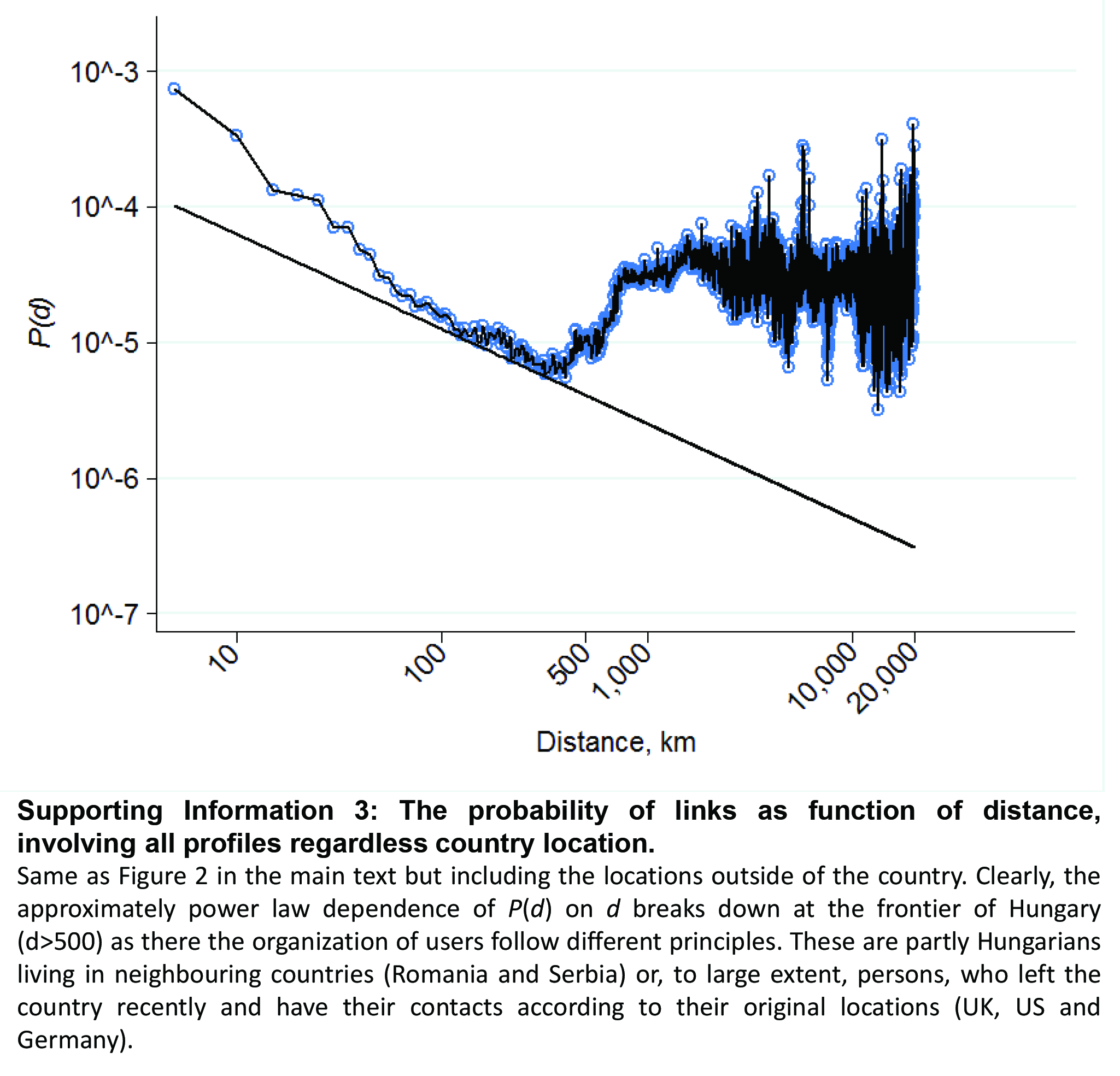

Supplement: S3 Fig — (TIF) [file pone.0137248.s003.tif]

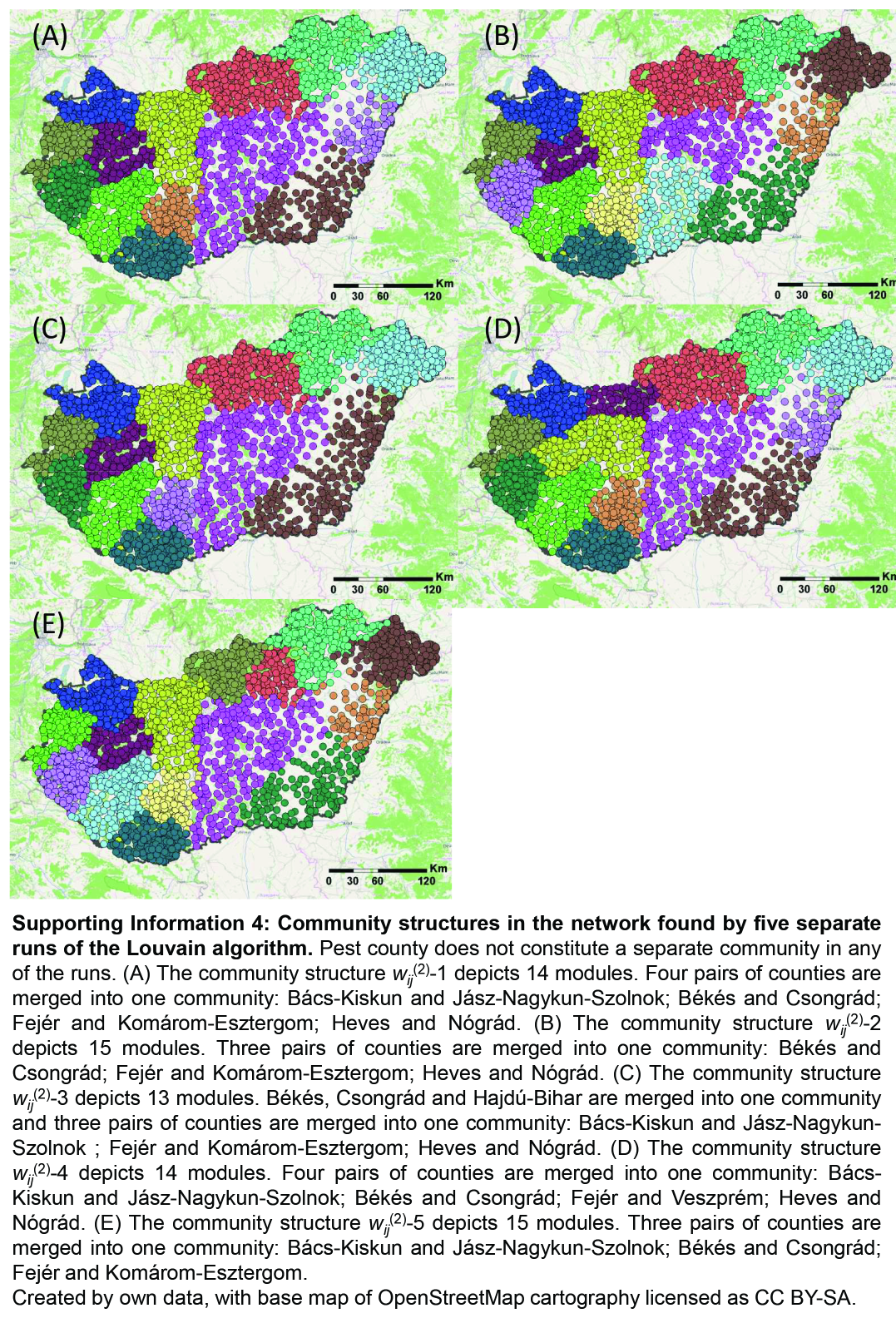

Supplement: S4 Fig — (TIF) [file pone.0137248.s004.tif]
